# Numerical investigation of elastic-plastic buckling performance of circular arched cellular steel beam using nonlinear finite element analysis method

**DOI:** 10.1016/j.heliyon.2024.e25292

**Published:** 2024-01-30

**Authors:** Besukal Befikadu Zewudie, Kefiyalew Zerfu, Elmer C. Agon

**Affiliations:** Faculty of Civil and Environmental Engineering, Jimma Institute of Technology, Jimma University, Ethiopia

**Keywords:** Elasto-plastic buckling, Cellular steel beam, Arched web post, Geometric imperfection, Buckling load

## Abstract

This study presents a numerical investigation of the in-plane elastic-plastic performance, post-buckling mode, and arched web-post shear resistance of a pinned end circular arched cellular steel beam using ABAQUS nonlinear finite element analysis package. The trustworthiness of the finite element analysis results was confirmed by comparing them to the existing experimental investigation results. The main study parameters, such as the effect of a rise-to-span ratio, the radius of curvature, the impact of opening, the types of loading on elastic-plastic performance, and the buckling mode of an arched cellular steel beam, were investigated. Furthermore, the arched web-post finite element model was proposed and the shear resistance of the arched web-post was investigated. Also, the appropriateness of the currently existing different web-post shear resistance analysis approaches was reviewed and evaluated in determining the shear resistance of arched web-posts. The results showed that the web post-structural stiffness of a circular arched cellular steel beam was improved as the rise-to-span ratio increased under the mid-span concentrated load regardless of the rise-to-span ratio. However, under uniformly distributed vertical load, increasing a rise-to-span ratio beyond 0.35 or 140° subtended angles reduces the stiffness of circular arched cellular steel beams. The web post shear resistance analyzing approaches proposed by Panedpojaman et al. and SCI P-100 overestimate and yield unsafe results in determining the web-post shear resistance of arched web post cellular steel of low rise-to-span ratio.

## 1 Introduction

In recent years, the utilization of steel sections with web opening such as castellated, cellular and sinusoidal steel beams are increasing. This is mainly due to its design and construction benefits such as an improved flexural stiffness, reduced weight per unit length, and construction advantages to run utilities through the openings. The evolution of fabrication technology, the aesthetical and mechanical performances extends utilization of cellular steel beams in to the forms of arched structural member in various modern building and bridges, especially as the best solution for long span arched roof systems [1]. Arched cellular steel beams are made from the parent hot rolled I-section by cutting it into two semi-circular tee sections, then curved at the required radius and welded together to form deeper, stiffer, and stronger arched cellular I-section steel beams [1,2].

Arched Structural members predominantly subjected to a combination compression and bending moment under general loading condition. An arch members that is adequately braced laterally is prevented from its out-of-plane failure. However, laterally unrestrained arched beams may suddenly fail in out of the plane mode during loading. Many of previous researches mainly focused on the in-plane and out-of-plane stability of solid web arched steel beams subjected to combined bending and compression actions [[3], [4], [5], [6], [7], [8], [9], [10], [11], [12]]. The in-plane strength of a steel arch depends upon various factors, such as the support stiffness, rise-to-span ratio, buckling behavior, yielding, initial curvature, included angle, slenderness ratio, residual stresses, initial in-plane geometric imperfections, and loading conditions [3]. The column-beam and classical buckling theory of analyzing in-plane strength of arched steel member does not consider the significant effect of pre-buckling deformation, and the nonlinearity of stress distribution in arch member [4]. Hence [4,7], develops a lower bound interaction equation for determining the in-plane strength of arched steel structural member under combined bending and axial compression considering pre-buckling deformation, and nonlinearity of stress distribution along the length of arch.

Due to the continuous web openings, cellular beams behave differently from its parent solid web I-section members. Structural elements with continuous web openings are highly indeterminate, which do not lend themselves to a simple method of analysis [13]. [14] was the first authors who described the additional local failure modes such as the Vierendeel yielding, the web-post-buckling, and web post welding fracture specific to steel beams with repeating web opening. Afterwards, numerous research reports have been presented on the local failure modes of steel beams with repeating web openings [[15], [16], [17], [18]]. Investigated the web post failure mode, and the impact of different parameters on the web post resistance of cellular steel beam, and developed novel design equations for web post shear resistance of cellular steel beam. Another local failure mode of continuous web opening steel beam is Vierendeel failure mechanism. Vierendeel failure occurs due to the high shear transfer around the opening which cause plastic hinges around the opening [19,20]. [19,20], investigated the location of shear stress concentration around the opening and identified the effective length of the opening to be considered in determining the Vierendeel bending resistance. The inadequacy of web post length for welding, can cause rupture of the welded joint at the web of castellated and cellular steel beams. This failure mode can be avoided by using sufficient web post length [13,21].

Due to the above-mentioned local failure modes, the design procedures and the in-plane strength of the beams with repeating web opening is quite different from the parent solid web I-section profile. The shape and size of opening also have a significant impact on the strength and behavior of the beam. Numerical investigation by [22] revealed that stress concentration around circular web opening is less when compared to the hexagonal web opening of the same area. As the number of web openings increases the ultimate strength and stiffness of the beam with opening decreases [23]. The provision of transverse stiffeners and ring stiffeners around the opening edge improves the buckling resistance of the cellular beam. When it comes to stiffeners comparison, transverse stiffeners are structurally more efficient than ring stiffeners around opening in resisting buckling of the cellular beam [23]. The presence of web holes reduces the axial strength in cold frame steel structures and concluded that edge stiffened web holes have an improved axial strength [24,25]. Furthermore, the web crushing and crippling mode of failure is significant in steel beam consisting of web opening as compared to beam without web opening. Researches showed that the provision of edge-stiffened holes increases the web crippling strength [26,27].

Numerical analysis by Ref. [28] investigated the effect of the web opening near to beam-column joint and the study revealed that edge openings close to beam-column joint connections have little effect on the strength of the connection. [29]Investigated sensitivity of the out-of-plane buckling of the freestanding roller bent circular steel arch to the initial geometric imperfection and residual stress, and found that the residual stress in hot rolled steel has more detrimental impact on the out-of-plane buckling than the residual stress pattern in roller bent steel arches.

Despite several types of research reported on the in-plane and out-of-plane structural behavior of solid web arched I-section steel and straight cellular steel beams, limited studies were reported on the behavior of arched cellular steel beams.

Moreover, design codes such as the European Standard (EC-3) [30], American National Standard Specification for Structural Steel Buildings [31], and American Iron and Steel Institute (AISI) [32] do not cover the design procedures for the in-plane and out-of-plane structural stability of circular arched cellular steel beams. The only existing experimental investigation on the arched cellular steel beams reported by Ref. [33], on the structural behavior of arched cellular steel beams which cover only three models under mid-span point loads with a limited range of testing parameters. Also, the study presented by Ref. [34] emphasizes on the effect of arch axis and end support types on load bearing strength of arched cellular steel beams. Hence, a detail nonlinear finite element based numerical investigation is required to understand the in-plane elastic-plastic buckling performance, post-buckling behavoir and arched web shear resistance under wide rage of parameters. This would provide a significant contribution to the understanding of the structural behavior of arched cellular steel beams under different scenarios and enrich databases for further study on the topic.

Therefore, this study presents a detailed nonlinear Finite Element Analysis (FEA) method of modeling and analysis for circular arched cellular steel beams and isolated arched web posts. In finite element analysis, elastic-plastic behavior of material, initial geometric imperfection, and second-order effect due to large deformation were included. The reliablity of FEA results confirmed by compering it to the existing experimental results. Furthermore, a parametric investigation into the impact of the rise-to-span ratio, the radius of curvature, the impact of web opening, and the types of loads on structural performance and post-buckling behavior of shallow and deep circular arched cellular steel beams were revealed in this investigation. Also, the shear resistance of arched web posts was investigated, and the appropriateness of the existing web post shear analyzing approaches for arched web posts was evaluated in this study.

## 2 Finite element analysis program

In this section, the detailed Finite Element Analysis (FEA) program is presented. The study was conducted using ABAQUS finite element software for the modeling and analysis of the test sample. Linear true stress-strain relation between points in the plastic region for the material behavior was assumed. In addition, Von-Mises yield criterion and isotropic hardening rule was utilized for the analysis. The inclusion of initial geometric imperfection on basis of estimated scale factor assumed. However, the residual stress due to manufacturing and curving was not considered in this study.

### 2.1 Material properties

For the parametric investigation of this study, the mechanical properties of steel section such as ultimate stress (fu), elastic modulus (Es), yield stress (fy), and the corresponding yield strain ($\varepsilon_{s}$) were adopted from the experimental tensile coupon test results reported by Ref. [11]. The bi-linear stress-stain curve of web and flange, extracted from tensile coupon tests were depicted in Fig. 2 (a) - (b), respectively. Poisson's ratio in the elastic region and ultimate strain hardening ensuring ductility were set to 0.3 and 0.023, respectively. Tensile coupon test results of the web and flange were described in Table 1. The plastic stress-strain matrices of equation given in Eq. (1) [35] was used in the finite element modelling to specify the plastic behavior of the material. Elastic-plastic analysis that uses von-Mises yield surface criterion was implemented in this study.

(1) $$\left\{{d\sigma }_{ij}\right\}=E\left[D^{p}\right]\left\{{d\varepsilon }_{ij}\right\}=2\left(1+v\right)G\left[D^{p}\right]\left\{{d\varepsilon }_{ij}\right\}$$

Where, {σ}, {ε} are the column matrices of stress $\sigma_{ij}$ and strain $\varepsilon_{ij}$ respectively, and $\left[D^{p}\right]$ represents 6 × 6 plastic stress-strain matrix, which explicitly expressed in Ref. [35]. Other notations such as E, G, and *v* are elastic modulus, the shear modulus, and Poisson's ratio, respectively.

**Table 1:** Mechanical properties of steel used for parametric investigation [11].

Plate origin	Plate thickness (mm)	Yield Stress, fy (MPa)	Elastic modulus E_s_ (GPa)	Ultimate Stress, fu (MPa)
Web	8	315	213	430
Flange	12	285	202	440

### 2.2 Test specimen

**Fig. 1:**
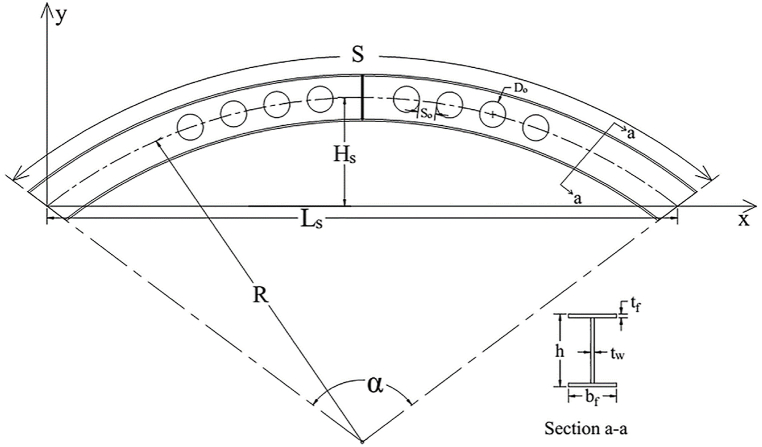
The dimensional components of the circular arched cellular beam.

For this investigation, I-section profile, which satisfy the class-1steel section criteria according to [37] was used. The section profile selected for the test sample can be used for medium-to-high load-bearing structures in actual engineering practices. The cross-sectional dimensions of the test sample beam are $h\;x\;b_{f}\;x\;t_{w}\;x\;t_{f}=250\;x120\;x\;8\;x12mm$, in which $h,b_{f},t_{w},and\;t_{f}$ are the overall height, flange width, web thickness and flange thickness, respectively. The geometry of the circular arch axis curve parameters shown in Fig. 1 was computed using Eq. (2) - Eq. (4) [36]. The dimensional properties of the circular arched cellular steel beam utilized for the finite element analysis were depicted in Table 2.

**Fig. 2:**
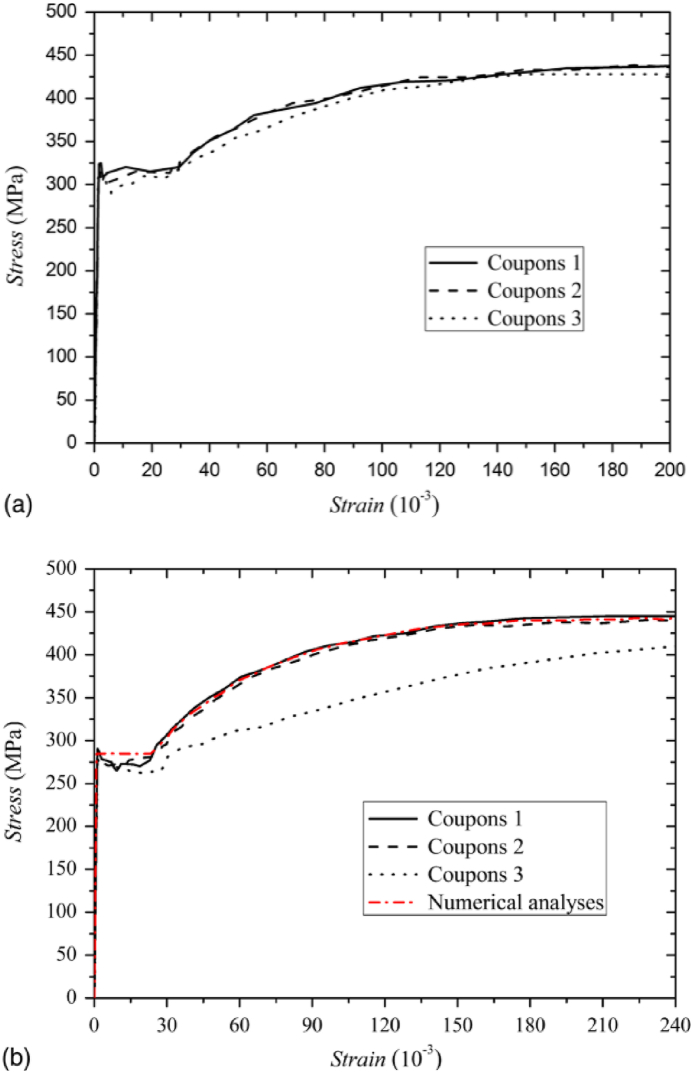
Bi-linear stress-strain curves for (a) web and (b) flange plate [11].

**Table 2:** Samples and their respective parameters.

Specimen	(H_S_/L)	α**(**deg.)	R (mm)	Hs (mm)	Do (mm)	Ls (mm)	Load case
SCC-1	0.099	45	3280.3	247.5	160	2500	MSPL
SCC-2	0.133	60	2515.9	332.5	160	2500	MSPL
SCC-3	0.207	90	1768.4	517.5	160	2500	MSPL
DCC-1	0.283	120	1458.0	707.5	160	2500	MSPL
DCC-2	0.35	140	1330.4	875.0	160	2500	MSPL
DCC-3	0.419	160	1269.6	1047.5	160	2500	MSPL
DCC-4	0.5	180	1250.0	1250.0	160	2500	MSPL
SCC-3000	0.207	90	2122.1	621.0	160	3000	MSPL
DCC-3000	0.35	140	1596.4	1050.0	160	3000	MSPL
SWSCC-1	0.099	45	3280.3	247.5	_	2500	MSPL
SWSCC-2	0.133	60	3280.3	332.5	_	2500	MSPL
SWSCC-3	0.270	90	1768.4	517.5	_	2500	MSPL
SWDCC-1	0.283	120	1458.0	707.5	_	2500	MSPL
SWDCC-2	0.35	140	1330.4	875.0	_	2500	MSPL
SWDCC-3	0.419	160	1269.6	1047.5	_	2500	MSPL
SWDCC-4	0.5	180	1250.0	1250.0	_	2500	MSPL
SCC-1*	0.099	45	3280.3	247.5	160	2500	URDVL
SCC-2*	0.133	60	2515.9	332.5	160	2500	URDVL
SCC-3*	0.207	90	1768.4	517.5	160	2500	URDVL
DCC-1*	0.283	120	1458.0	707.5	160	2500	URDVL
DCC-2*	0.35	140	1330.4	875.0	160	2500	URDVL
DCC-3*	0.419	160	1269.6	1047.5	160	2500	URDVL
DCC-4*	0.5	180	1250.0	1250.0	160	2500	URDVL
SCC-3000*	0.207	90	2122.1	621.0	160	3000	URDVL
DCC-3000*	0.35	140	1596.4	1050.0	160	3000	URDVL
SWSCC*-1	0.099	45	3280.3	247.5	_	2500	URDVL
SWSCC*-2	0.133	60	3280.3	332.5	_	2500	URDVL
SWSCC*-3	0.207	90	1768.4	517.5	_	2500	URDVL
SWDCC*-1	0.283	120	1458.0	707.5	_	2500	URDVL
SWDCC*-2	0.35	140	1330.0	875.0	_	2500	URDVL
SWDCC*-3	0.419	160	1269.6	1047.5	_	2500	URDVL
SWDCC*-4	0.5	180	1250.0	1250.0	_	2500	URDVL

➢ Where, *SCC is shallow circular arched cellular steel beam, DCC is deep circular arched cellular steel beam, MSPL is mid-span point load, URDVL is uniform radially distributed vertical load, SWSCC-solid web shallow circular arch, SWDCC-solid web deep circular arch*.

The web post opening spacing S and opening diameter $D_{o}$ were chosen according to the BS EN 1993-1-1 [37]. To prevent pre-mature yielding around the opening due to high shear stress concentration, the opening diameter and web post length of the cellular beam should be within $1.25<h/D_{o}<1.75$ , and $1.08<S/D_{o}<1.5$ range, respectively. To meet the above criterion, $D_{o}=160\;mm,s=220mm,\text{and}\;\text{web}\;\text{post}\;\text{width}\;S_{o}=60mm$ were used in this study.

(2) $$y=H_{s}-R+\sqrt{R^{2}-{\left(\frac{L_{s}}{2}-x\right)}^{2}}$$

(3) Hs=R[1−cos(α2)]

(4) Ls=2R*sin(α2)

Where, *S* is arch length, *H*_*s*_ is an arch rise, *L*_*s*_ is span length, *R* is the arch radius and αissubtendedangle.

### 2.3 Element type and mesh

Shell elements from the ABAQUS library make it easier to simulate curved shells that are subjected to high translation, rotation, and stress levels as well as nonlinear material response [38]. For this study, three-dimensional linear geometric order shell element of a combined 4-node (S4R) and 3-node (S3R) of doubly curved thin shell with reduced integration numerical techniques to integrate various quantities over the volume of each element were used together. The size of the mesh elements affects the accuracy of the finite element model response such as buckling load, deformation, stress, and strain. The finer element size adds more nodes with a degree of freedom that can accurately capture structural response. However, it takes a longer time to compute. On the contrary, the coarse element mesh size gives the model too few degrees of freedom, it may produce wrong analytical results in a relatively short amount of time for solving. Therefore, until converged analysis results obtained, it is crucial to iteratively reduce the mesh element size from coarse to finer element size. Beyond optimum mesh size, further reducing of mesh size has little or no effect on analysis output rather than lengthening solving time. For this study 20 mm optimum mesh size discretize model into a shell element of a 1665 and with number of nodes 1819 gives converged results of Eigen buckling load, inelastic buckling load and deflection of the model.

### 2.4 Boundary condition and loading

**Fig. 3:**
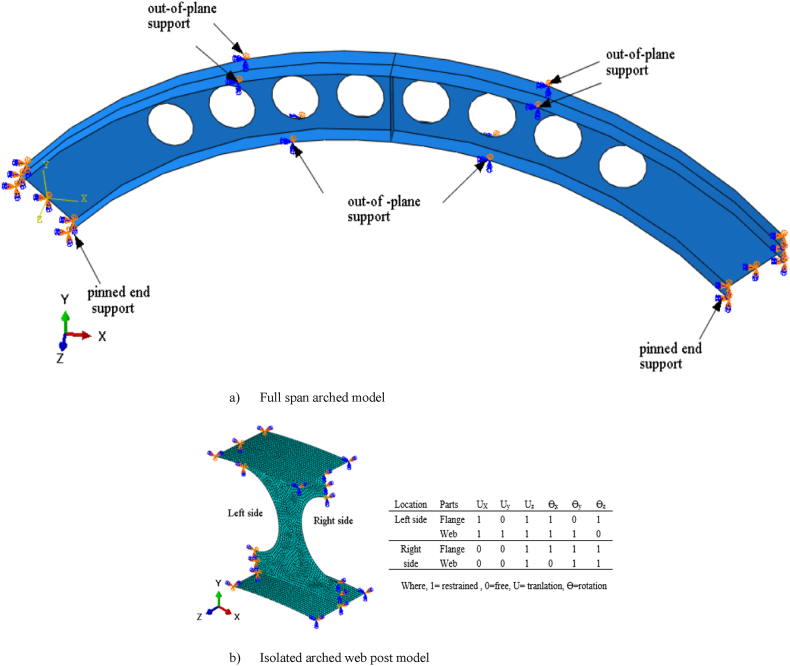
Boundary conditions of Finite Element Analysis.

The boundary conditions were adopted based on the existing experimental setup shown in Fig. 5. In addition to pinned end supports, out-plane supports provided at one-third of the sample span length to prevent premature lateral-torsional buckling as shown in Fig. 3 (a). The details of the boundary conditions are depicted in Table 3. The boundary conditions utilized in finite element modeling of the isolated arched web-post was given in Fig. 3 (b).

**Table 3:** Details for boundary conditions.

Support type	Ux	Uy	Uz	$\theta_{X}$	$\theta_{y}$	$\theta_{z}$
Pinned	1	1	1	1	1	0
Out-of-plane support	0	0	1	1	1	0

Where, *1=restrained, 0=free, U=translation & θ = rotation*.

Loading of the beam is dependent on the step analysis. In the first step, linear buckling analysis implemented by applying a unit load to obtain minimum eigenvalue, which is later used as reference load to calculate the critical non-linear buckling load. The critical buckling load obtained from linear buckling step was applied to the beam using general Static Riks method algorithm to calculate the true in-elastic buckling load of the circular arched cellular steel beam.

### 2.5 Geometric imperfection

**Fig. 4:**
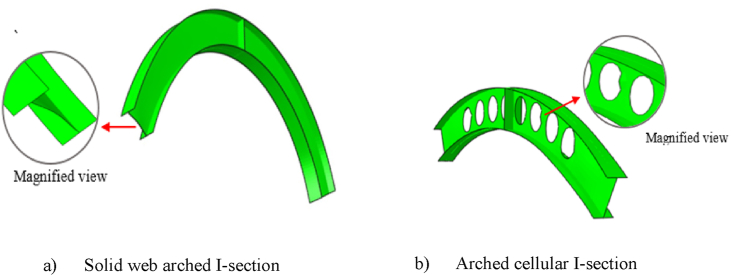
Initial geometric imperfection plot.

In the process of cutting, manufacturing, and transporting of the arched steel beam, initial out-of straightness is expected, and it is unavoidable. This geometric deviation significantly contributes to the initiation of buckling. To take the impact of geometric imperfection, two steps of finite element analysis were performed; first linear buckling analysis was undertaken to determine the lowest Eigen buckling mode shape which has strong coherence with an arch deflection at elastic buckling. Thus, introducing initial geometric imperfection with scale factor of S/1000 to lowest Eigen buckling mode shape obtained from linear buckling analysis provides a good estimation to define initial geometric imperfection in the second nonlinear buckling analysis [11,29,39,40]. Fig. 4 (a)-(b) indicates a finite element model with initial geometric imperfection.

**Fig. 5:**
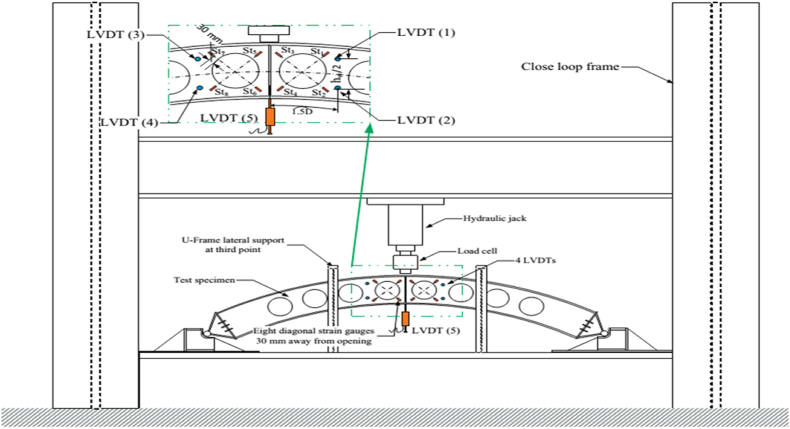
Experimental setup and instrumentation [33].

### 2.6 Analysis step

The main objective was to find the in-elastic ultimate buckling load with post-buckling behavior. Therefore, load-controlled analysis step was implemented in this study.

To determine the ultimate inelastic buckling load and complex post-buckling behavior of arched cellular steel, two-step analysis approaches were used. First, the linear buckling analysis implemented to extract eigenvalue with linear buckling mode. This step used linear perturbation analysis step to provide simple linear buckling response. To obtain the complex nonlinear response of the member by taking in to account of the effect of material nonlinearity, initial geometric imperfection, second order effect due to large deformation nonlinear buckling analysis step was required. In the second step nonlinear Static Riks analysis procedure was used to find inelastic load-deflection and nonlinear buckling response by taking into account the effects of geometric imperfection and material nonlinearity. The Static Riks method follows a linear buckling analysis step to compute load proportionality factor by using the lowest Eigenvalue as reference load vector. The modified Static Riks algorithm simultaneously solves for loads and displacements using arch length method procedure in preference to Newton–Raphson to solve the non-linear equilibrium equations. According to past studies, the arc-length approach process follows the proper equilibrium path for nonlinear problems that arise from significant changes in geometry or material behavior when buckling instability and softening predominate [[41], [42], [43]]. This method traces the solution's progress in load-displacement space along a static equilibrium path. The Static Riks analysis step approach offers load proportionality factor (λ) as part of the solution. The elastic buckling load defined in the Riks step is referred to as a reference load.

Ultimate inelastic buckling load ($P_{u}$) can be related to load proportionality factor and to the reference load as given by Eq. (5).

(5) $$P_{u}=\lambda P_{ref}$$

Where, $P_{ref}$ is the reference load vector, and λ is the load proportionality factor. At each increment, the load proportionality factor is calculated as part of the solution [43].

### 2.7 Validation of the proposed FE model

The reliability of the proposed finite element model results were validated by comparing them against the existing related experimental work reported by [33]. [33] Investigated full-scale experimental tests on the structural behavior of an arched cellular steel beam with corresponding failure mode under point load applied at the mid-span of the arch using a hydraulic jack of 1000 kN capacity. The load on the beam was gradually applied in increments of approximately 5 kN steps until web post-buckling was initiated and visible [33]. The lateral displacement of the web and the vertical displacement of the tested specimens were measured using linear variable differential transformers (LVDTs) at a specific location, the experimental setup is depicted in Fig. 5. Geometrical and cross-sectional details of the experimental specimens conducted by [33] are tabulated in Table 4.

**Table 4:** Dimensional properties of experimentally tested arched cellular steel beam specimens by [33].

Specimens	h(mm)	$t_{w}\;\left(mm\right)$	$b_{f}\;\left(mm\right)$	$t_{f}\;\left(mm\right)$	Ls (mm)	S (mm)	Hs (mm)	Α (deg.)	Do (mm)	Yield Stress, fy (MPa)		Ultimate Stress, fu (MPa)		Modulus of Elasticity, Es (GPa)	
										Web	Flange	Web	Flange	Web	Flange
B-1	170	4	120	8	2451	2590	472	60	–	242	233	290	315	205	199
B-2	240	4	120	8	2451	2590	538	60	160	242	233	290	315	205	199
B-3	240	4	120	8	2651	2590	699	60	160	242	233	290	315	205	199
B-3	240	4	120	8	2032	2590	484	60	160	242	233	290	315	205	199

**Fig. 6:**
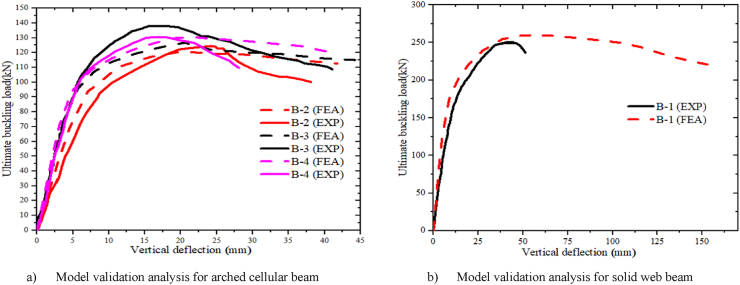
Model validation study.

Fig. 6 and Table 5 illustrate the comparative study of the proposed FE-analysis results against the related existing experimental work. As can be seen from Fig. 6 (a) and (b) the buckling load versus in-plane deflection curve extracted from FEA agreed well with the experimental test reported by [33]. The Ultimate inelastic buckling load and the associated mid-span in-plane vertical deflection obtained from FE-analysis and experimental test results depicted in Table 5 show that the ultimate buckling load and in-plane deflection results obtained from FEA model is reasonably a match with the experimental one with slight differences. The slight differences between the FEA model and the experimental results can be due to the difference between the fully pinned boundary condition used for finite element modeling and the pivotal boundary condition of the experimental model, the difference of real material behavior and ideal bilinear property used for modeling, inaccuracy of geometric imperfection scaling, and unconsidered residual stress. Therefore, the FEA model proposed is capable of replicating the experimental works to investigate the load-deflection and post-buckling response of arched cellular steel beams with good accuracy.

**Table 5:** Comparison of buckling load and in-plane vertical deflection, between Experimental and FEA results.

Specimens	Ultimate buckling load (P) (kN)				In-plane vertical deflection (U) (mm)			
	$P_{\mathrm{Exp}}$	$P_{FEA}$	$P_{\mathrm{Exp}}/P_{FEA}$	Deviation(%)	$U_{\mathrm{Exp}}$	$U_{FEA}$	$U_{\mathrm{Exp}}/U_{FEA}$	Deviation(%)
B-1	253	259.95	0.97	2.67	44	52.6	0.84	16.34
B-2	124	120.77	1.02	2.36	24	20.11	1.19	16.20
B-3	137	126.69	1.08	7.52	18	20.58	0.87	14.33
B-4	130	130.31	0.99	0.23	17	19.28	0.88	13.41

### 2.8 Parametric study

Thirty-two samples of circular arched cellular steel beams were analyzed, to examine the effects of the rise-to-span ratio, radius of curvature, and the web openings on the in-plane inelastic buckling strength and buckling mode of circular arched cellular steel. The structural behavior of the test samples were examined under uniformly distributed vertical and mid-span concentrated static load. Furthermore, seven arched web posts at different degree of curvature were analyzed to investigate the buckling shear resistance of arched web post. The test samples considered in this investigation were grouped into two classes depending upon the degree of curvature as shallow circular cellular arch, for samples with subtended angle α ≤ 90° and deep arched cellular steel beams, test samples with subtended angle α > 90°. Samples SCC-1 toDCC-4 have the same span length, but varied in rise-to-span ratio and the radius of an arch as depicted in Table 2. To examine the impact of radius of curvature, samples of the same span-to-rise ratio but only different in span length were considered. To figure out the effect of repeated circular web opening on inelastic buckling capacity and buckling modes, the samples of solid webs of the same cross-sectional dimensions were compared to the corresponding arched cellular steel beams of the same span length, rise -to-span ratio and arch radius as described in Table 2.

## 3 Result and discussion

Under this section, an extensive study results presented for a cellular arched steel beams under mid-span concentrated load and uniform distributed load. Also, the finite element model of arched web post are presented for analyzing arched web post shear resistance and compared to different existing analytical approaches used for determining web post shear resistance.

### 3.1 Effect of the rise-to-span ratio

**Fig. 7:**
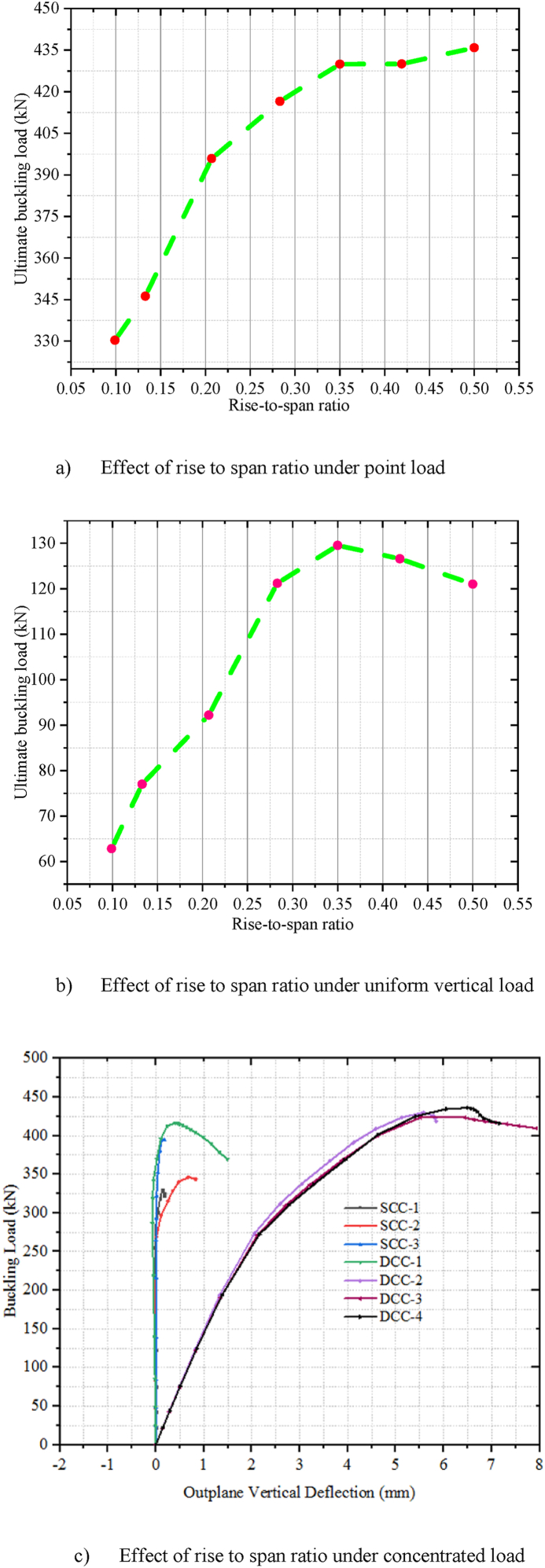
FEA analysis results for the effect of rise to span ratio.

The specimens SCC-1-DCC-4 and SCC-1*- DCC-4* described in Table 2 are the test samples of circular arched cellular steel beam covering from shallow to deep arched beam under concentrated mid-span concentrated load and uniform radially distributed vertical load, respectively. The subtended angle of the the test samples ranges from 45° to 180°. All the specimens have the same cross-sectional and material properties. However, the rise-to-span ratio was altered with subtended angle as the main study parameter. The ultimate buckling load that causes web buckling increases with the rise- to-span ratio, due to the effect of arch action under concentrated load, as shown in buckling load to in-plane vertical deflection analysis results in Table 7 and Fig. 7(a). The arch action enhances the internal resistance of the arched beam as rise-to-span ratio increases under mid span concentrated load. However, under uniform radially distributed vertical load, the stiffness of the arch due to the impact of arch action is not similar to the same tesr models under mid-span concentrated load. In-elastic buckling load versus in-plane deflection depicted in Fig. 7(b) revealed that ultimate buckling load-carrying capacity of arched cellular steel beam increases with the rise-to-span ratio up to DCC-2 of the test sample with the subtended angle of $140^{0}$ and the rise-to-span ratio 0.35.Further increase of the rise-to-span ratio beyond 0.35 reduces the stiffness of the arch under uniformly distributed vertical load. Table 8 depicts the percentage of increment of buckling load with the rise-to-span ratio of the test models under uniform vertical load.

**Table 6:** Buckling shear resistance of isolated arched web post FEA results and buckling shear resistance of experimental web post-test models.

Isolated arched web post shear resistance (V_VT_) (kN)				
specimen	FEA model result	EXP. test result [33]	VVT,EXPVVT,FEA	Difference in percentage
B-2	50.97	53	1.04	3.83
B-3	56.55	59	1.12	4.15
B-4	54.15	55	1.02	1.54

**Table 7:** FEA analysis result for arched cellular under point load.

Specimen	$P_{u}$ (kN)	$U_{\max}$ (mm)	Increment (%)	Failure Modes
SCC-1	330.32	17.58	–	WPB
SCC-2	346.23	18.34	4.59	WPB
SCC-3	395.89	25.38	12.54	WPB
DCC-1	426.75	24.34	5.00	WPB
DCC-2	429.97	22.56	3.04	WPB
DCC-3	430.01	29.49	0.23	WPB
DCC-4	435.89	35.08	1.34	WPB

**Table 8:** FEA analysis result for arched cellular under uniform load.

Specimens	$P_{u}$ (kN)	$U_{\max}$ (mm)	Increment (%)	Failure Mode
SCC-1*	62.84	21.96	–	WPLTB
SCC-2*	77.01	23.32	18.4	WPLTB
SCC-3*	92.19	18.52	16.46	OPB
DCC-1*	121.18	15.82	24	OPB
DCC-2*	129.52	12.12	6.43	OPB
DCC-3*	126.29	12.25	−2.49	OPB
DCC-3*	121.01	10.94	−4.18	OPB

Where, P_u_ is ultimate load, U_max_ is maximum mid-span vertical deflection and WPB is web post-buckling, WPLTB is web post lateral torsional buckling, and OPB is out of plane buckling.

### 3.2 Effect of the radius of curvature

**Fig. 8:**
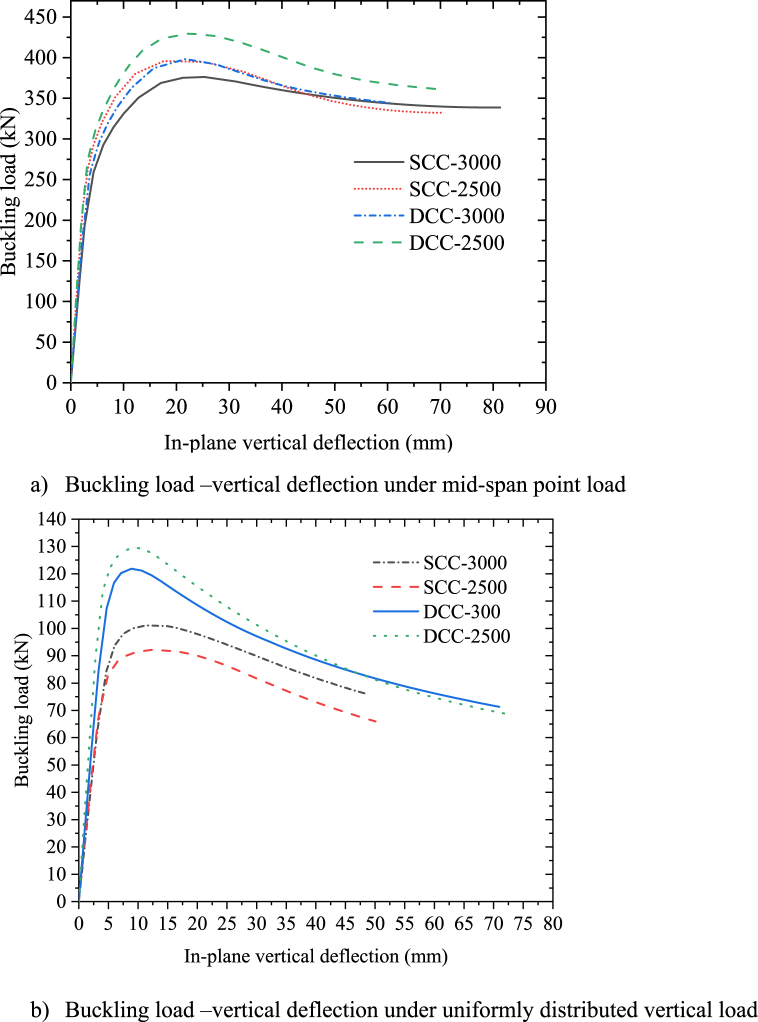
Effect of radius of curvature for deep and shallow arch.

To examine the effect of radius of curvature on the internally induced stress of symmetric circular arched cellular steel structure, four test samples (SCC-2500, SCC-3000, DCC-2500, and DCC-3000) were used. These samples have an identical cross-section, material property, rise-to-spanratio and subtended angle, but different in span length as shown in Table 2. FEA results of inelastic buckling load to in-plane deflection curve of Fig. 8(a) indicates that irrespective of the degree of curvature of an arch, increasing the radius of curvature also increases the horizontal shear force which induces compression stress over web post under mid span point load. Due to this mechanics under mid-span concentrated load, increasing span length of an arch of the same rise-to-span ratio reduces the in-plane buckling resistance of circular arched cellular steel beams. But, under uniform radially distributed vertical loads, these test models’ analysis result shows tha the effect of radius of curvature depends upon the degree of curvature. For shallow circular cellular arches, increasing the span length of the same arch of the same rise-to-span ratio reduces the induced horizontal shear stress on the web which causes web post-buckling. Therefore, longer span shallow circular arched steel beams of the test model SCC-3000 is stiffer than short span SCC-2 of the same rise-to-span ratio. In reverse, increasing radius curvature for deep arched circular cellular steel beams reduces the in-plane inelastic buckling capacity as shown in Fig. 8(b).

### 3.3 Impact of the web openings

**Fig. 9:**
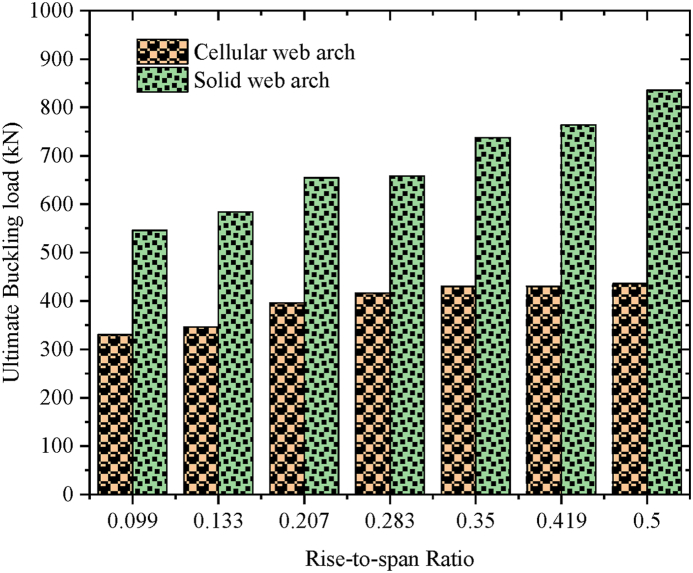
Under mid-span concentrated load.

**Fig. 10:**
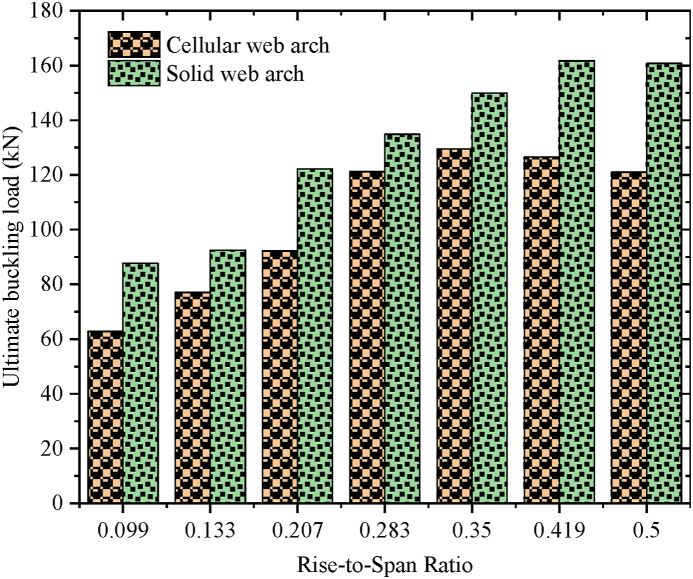
Under uniform radially distributed vertical load.

To investigate the effect of web opening on arch stiffness and post-buckling behavior, cellular arched specimens were compared to the corresponding solid web arches of the same cross-section and arch geometry as described in Table 2. The comparison of the test models performed under static mid-span concentrated and uniform radially distributed vertical load. As expected, on average, due to the presence of repeated web opening, the structural stiffness of circular arched cellular steel beams is reduced by 60.5 % and 82.75 % compared to solid web arches under mid-span concentrated load and uniform radially distributed vertical load, respectively, as shown in Fig. 9, Fig. 10.

The three-dimensional simulated failure modes also indicates that the presence of repeated web openings alters the internal stress distribution in the arch that varies with the rise-to-span ratio. Under mid-span point load, shallow cellular arches SCC-1 and SCC-2 fail in web post-buckling of the web near the load application point as shown in Fig. 16 (a) and (b), but solid web arches SWCC*-1 and SWCC*-2 buckle in symmetric in-elastic out-of-plane mode as shown in Fig. 20 (a). Under uniformly distributed load, shallow cellular circular arches buckle in lateral-torsional web buckling of the web near the support, as depicted in Fig. 17. However, the corresponding solid web arches SWCC-1 and SWCC-2 buckle due to the yield of the outside flange near the end support as shown in Fig. 20(b).

Deep circular solid arches SWDCC-1 and SWDCC-2 buckle in elastic symmetric out-of-plane deformation as depicted in Fig. 21 under mid-span point load. Under uniform vertical load, test specimens’ models of solid web SWDCC*-1 and SWDCC*-2 buckle in antisymmetric inelastic out of plane buckling as shown in Fig. 22. Generally, these results shows that the introduction of repeated web opening of arches affects stiffness, internal stress distribution, and the buckling failure modes of steel arches.

### 3.4 Failure modes in circular arched cellular steel beam

Under this subsection, the failure modes observed from the finite element analysis results of circular arched cellular steel beam under mid-span concentrated and uniform radially distributed vertical load is discussed. To support the relevance of failure mechanism obtained from finite element analysis, previous experimental works on buckling modes of arch steel structures and failure mechanism of cellular steel beams have been reviewed [14,44,45]. For the current study, web post-buckling (WPB), web post lateral torsional buckling and out-of-plane buckling modes of arched cellular steel beams are presented and compared to failure mechanisms of solid web arch steel beam.

#### 3.4.1 Web post-buckling

For arched cellular steel beams of restricted web opening spacing and diameter according to BS EN 1993-1-1[37], the web-post-buckling is the predominant failure modes observed from finite element analysis, regardless of the arch degree of curvature under mid-span point load as shown in Fig. 16 (a)–(c). The difference in the axial force between web openings induces horizontal shear (V_h_) on force on the web post, which causes diagonal compression and tensile stress on the web post as can be seen from 13 (a)-(c). Fig. 14(c) illustrates how the compression load twists the web post along its vertical axis; this failure mode is known as web-post buckling.

Despite the predominant occurrence of web post buckling mode on arched cellular steel beams under mid-span point load, there is no general consensus about the analytical models for computing horizontal shear force (V_h_) and vertical shear resistance (V_v_) of web post for arched cellular steel by taking the significant effect of uneven internal force distribution, impact of the rise-to-span ratio and the loading types. Rather, the analytical model approaches developed for straight cellular steel beam web post is used in determining web post shear resistance of arched cellular steel structure members.

In this section, arched web post was modeled and analyzed using finite element analysis to evaluate the suitability of different existing analytical model used to calculate the web post shear resistance of arched cellular steel. The isolated web post geometry used in this section was the same as of the full span arch web post where web post-buckling noticed under mid-span point load. The isolated arched web post test sample models wererefined from the full span arch samples presented in Table 2 was used in this section.

In finite element modeling of isolated arched web post in this section, the same material property, initial geometric imperfection, and analysis step as of the full span arched cellular test models described Section 2 was implemented.

Two analyzing steps were incorporated in analyzing isolated arched web post shear resistance. First linear buckling analysis was performed to obtain the lowest Eigen value with mode of buckling then the buckling mode of lowest Eigen value used by integrating scale factor for initial geometric imperfection with material nonlinearity, and effect of second order in the second nonlinear analysis step to obtain vertical shear resistance of arched web post. Fig. 14 (a)–(c) depicts un-deformed, the linear and nonlinear buckling of isolated arched web post. The boundary condition of the isolated arched web post model implemented in this study was shown in Fig. 3 (b).

The validity of isolated arched web post shear resistance results obtained from the finite element analysis were verified by comparing it to the experimental full length arched cellular steel beams web post shear resistance presented in existing literature [33]. As it can be seen from Table 6, the finite element analysis results of web-post shear resistance results matches to the experimental results.

In this subsection, different analytical models that are proposed to determine the web-post shear resistance of straight cellular steel beams were reviewed and compared to the arched web-post shear resistance extracted fromthe FEA. The suitability of these different approaches in determining the web shear resistance of arched web posts were also evaluated. The SCI P-100 [16] web post shear resistance determining approach, the Lawson et al. [15] andPanedpojaman et al. [46] proposed effective web post length along with BS EN 1993-1-1 [37,47], and the web post shear resistance analyzing approach proposed by Figueiredo et al. [45] have been reviewed and their appropriateness in determining web post shear resistance of arched web were evaluated.

**Fig. 11:**
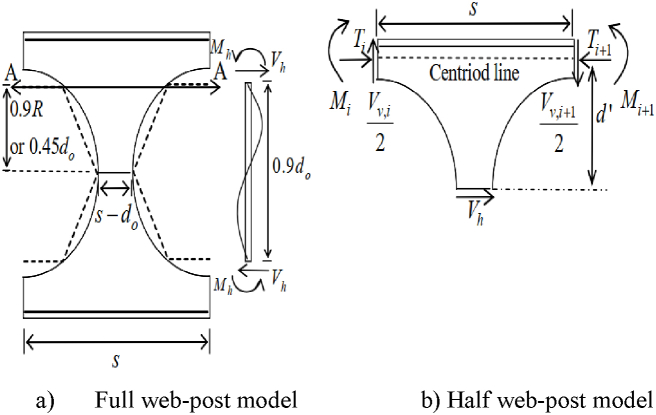
Typical web post modelling for internal action [16].

In 1990, Steel Construction Institute (SCI) of the United Kingdom analyzed web post-buckling by proposing typical web post modelling [16], in which the perforated web is considered as an isolated column as an equal and opposite shear and moment acted upon the web post as shown in Fig. 11 (a)-(b). According to this approach, the maximum web-post moment and shear capacity of web-post primarily dependent on the web thickness (t_w_) and the ratio of web opening spacing to opening diameter ($s/d_{0}$) [16].

The Steel Construction Institute publication number 100 [16] proposed that the maximum horizontal shear resistance of the web post can be determined from maximum allowable web moment (M_max_) as given in Eq. (6) at critical section A-A shown in Fig. 11 (a), where, $M_{e}$ is the ultimate elastic moment capacity of the web, which is the product of section modulus of the web and yield strength of the steel (f_y_) as expressed in Eq. (7). The ratio of maximum allowable web post moment (M_max_) to the elastic moment capacity of the web post (M_e_) given in Eq. (8). Where, C_1,_ C_2,_ and C_3_ are constants which are the function of diameter of opening $\left(d_{o}\right)$ and web thickness $\left(t_{w}\right)$ ratio as given in Eq. (9)-Eq. (11).

(6) $$M_{\max}=0.9R*V_{h}$$

(7) $$M_{e}=\frac{t_{w}\left(S-d_{o}+0.564{d_{o}}^{2}\right)}{6}f_{y}$$

(8) $$\frac{M_{\max}}{M_{e}}=\left[c_{1}\left(\frac{S}{d_{o}}\right)-c_{2}{\left(\frac{S}{d_{o}}\right)}^{2}-c_{3}\right]$$

(9) $$c_{1}=5.097+0.1464\left(\frac{d_{o}}{t_{w}}\right)-0.00174{\left(\frac{d_{o}}{t_{w}}\right)}^{2}$$

(10) $$c_{2}=1.41+0.0625\left(\frac{d_{o}}{t_{w}}\right)-0.000683{\left(\frac{d_{o}}{t_{w}}\right)}^{2}$$

(11) $$c_{3}=3.645+0.0853\left(\frac{d_{o}}{t_{w}}\right)-0.00108{\left(\frac{d_{o}}{t_{w}}\right)}^{2}$$

**Fig. 12:**
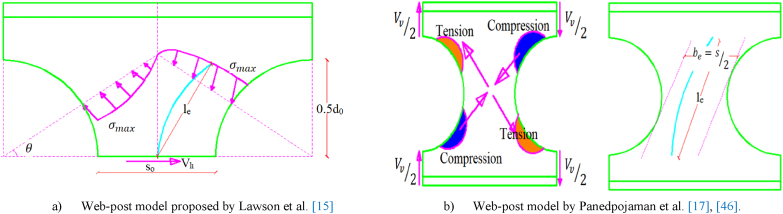
Strut model for web post buckling modelling.

**Fig. 13:**
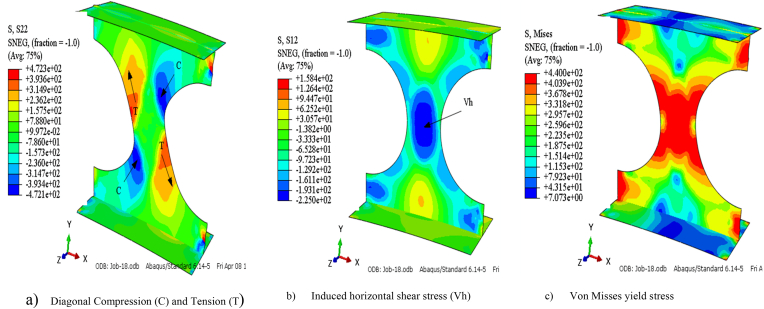
FEA model stress distribution on isolated arched web post (SCC-1 test model).

The modified approaches for analyzing web-post shear resistance is the strut analogy. According to this method, the compression (strut) twists web-post over its vertical axis, acts diagonally as shown in Fig. 12 (a)–(b) and Fig. 13 (b). Due to uneven distribution of stress around the web opening, establishing the precise effective buckling length (l_e_) of the web-post is challenging. Lawson et al. [15] and Panedpojaman et al. [46] proposes two different analytical equations to determine the effective buckling length (l_e_) for symmetric web post of circular openings.

**Fig. 14:**
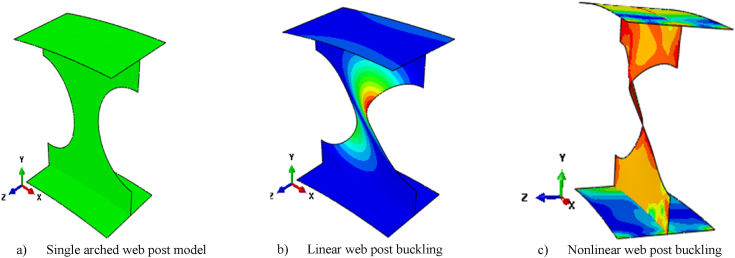
Linear and non-linear web post buckling of isolated arched web post (from SCC-1 test model).

Lawson et al. [15] developed effective buckling length ($l_{e,\text{Lawson}\;}$) of single web post geometry as given in Eq. (12) by considering that the effective width ($b_{e})$ of web post is equal to the half of the web post width $(s_{o}/2)$. The web post buckling model proposed by Lawson et al. [15] is shown in Fig. 12 (a).

(12) $$l_{e,\text{Lawson}\;}=0.5\sqrt{{s_{o}}^{2}+{d_{O}}^{2}}\leq 0.7d_{o}$$

Later on, Panedpojman et al. [46], modified the Lawson et al. [15] Effective web buckling length model by considering the contribution of large area above the opening on the web post buckling resistance by proposing the coefficient of effective length (k) into Eq. (13). The effective web-post model of Panedpojaman et al. [46] is shown in Fig. 12 (b).

(13) $$l_{e,\text{panedpojaman}\;}=k*0.5*\sqrt{S^{2}-d_{o}^{2}}$$

Where, (k) is an effective buckling length factor and can be calculated as Eq. (14).

(14) $$k=0.9\left(\frac{d_{o}+s_{o}}{d_{0}}\right)\leq \min\left(1.15\frac{d_{o}}{H},1.15\right)$$

Where $d_{o}$ is opening diameter, $s_{o}$ is web post width, S is opening center to center spacing, and H is the overall depth of the section.

For the design purposes, the horizontal shear buckling resistance (Vh,r) of web post of straight cellular steel beam can be calculated using Eq. (15) according to BS EN 1993-1-1 [37,47]. The compressive strut (σ) acted up on the web can be estimated by Eq. (16), where χ is the buckling reduction factor for accounting the risk of flexural buckling as a function of non-dimensional slenderness ratio $\left(\bar{\lambda \;}\right)$ and imperfection factor (α). In this study, to compute the buckling reduction factor (χ) the equations given in Eq.(17), (18), (19), (20), (21) for a member under compression was adopted from BS EN 1993-1-1 [47]. Once the horizontal shear ($V_{hr}$) that causes web post-buckling obtained, the vertical shear resistance of the upper and lower tee can be related to horizontal shear resistance of web post as in Eq. (22). For symmetric cellular beams, the total vertical shear resistance $\left(V_{v,r}\right)$ of the web post computed as the sum of the upper and the lower tee section vertical shear resistance [46].

(15) $$V_{hr}=\sigma *b_{e}t_{w}$$

(16) $$\sigma =\chi f_{y}$$

(17) $$\chi =\frac{1}{\varphi +\sqrt{\varphi^{2}-{\bar{\lambda }}^{2}}}$$

(18) $$\varphi =0.5\left[1+\alpha \left(\bar{\lambda }-0.2\right)+{\bar{\lambda }}^{2}\right]$$

(19) $$\bar{\lambda }=\sqrt{\frac{f_{y}}{f_{e}}}$$

(20) $$f_{e}=\frac{\pi^{2}\;E}{\lambda^{2}}$$

(21) $$\lambda =\frac{\sqrt{12l_{e}}}{t_{w}}$$

(22) $$V_{v,r,U}=V_{v,r,L}=V_{hr}\frac{2*Y_{o}}{S\;}$$

In which, λ is the slendernes of web post, $f_{y}$ is the web post yield strength, $f_{e}$ is the.buckling stress acting over web post, S is the center to center spacing of the openings, $Y_{o}$ is the distance between centroid of the web post to the center of tee section and, $l_{e}$ is effective buckling length of the web post computed as proposed by Lawson et al. [15] and Panedpojamanet et al. [46]. The imperfection factor (α) = 0.49 for European buckling curve c was used for this study.

The other analytical approache evaluated in this investigation is the recent proposed plastic shear resistance formula presented by Figueiredo et al. [45]. This approach proposed the web post shear resistance determining formula as given in Eq. (23) considering full plastification of the section due to normal and shear stress interaction at critical distance from the web post center line.

(23) $$V_{\text{hr},\text{pl},\text{Figueiredo}}=\chi \;\mu f_{y}\frac{t_{w}{b_{\text{pl}}}^{2}}{\sqrt{3{b_{\text{pl}}}^{2}+16{y_{\text{pl}}}^{2}}}$$

Where, the $y_{pl}$ and $b_{pl}$ are the critical distance from the web post center line to the location where plastification occurs and web post width at point of plastification, respectively. The emperical equations given in Eq.(24), (25), (26), (27), (28) were proposed to calculate $y_{pl}$, $b_{pl}$, adjustment factor (μ) and buckling reduction factor (χ),respectively.

(24) $$y_{\text{pl}}=\frac{d_{o}}{2}\left[0.445{\left(\frac{S}{d_{o}}\right)}^{3}-2.578{\left(\frac{S}{d_{o}}\right)}^{2}+4.77\left(\frac{S}{d_{o}}\right)-2.475\right]$$

(25) $$b_{pl}=S-d_{o}\sqrt{1-\frac{4y_{pl}}{{d_{o}}^{2}}}$$

(26) $$\mu =1.198-0.42\frac{d_{o}}{H}+\frac{S}{5d_{O\;}}\;\text{for}\;\frac{S}{d_{o}}<2$$

(27) $$\chi =\frac{\alpha }{{\lambda_{o}}^{\beta }}\leq 1.0\;for\;\lambda_{0}\geq 1.0$$

(28) $$\chi =\left\{ \begin{array}{c} \frac{\alpha }{{\lambda_{o}}^{\beta }}\leq 1.0\;\text{for}\;\lambda_{0}\geq 1.0\;\\ \gamma \varepsilon^{{\lambda_{o}}^{\eta }}\;for\;\lambda_{0}<1 \end{array}\right.$$

Where, $\lambda_{0}$ is non-dimensional slendernes ratio of web post computed as given in Eq. (29) and the coefficient values of α,β,γ,ε,andη are provided as the function of the ratio of $\frac{d_{o}}{H}$ and $\frac{S}{d_{o}}$ [45].

(29) $$\lambda_{o}=\frac{\lambda }{\pi }\sqrt{\frac{f_{y}}{E}}=\sqrt{\frac{3\left(S^{2}-{d_{o}}^{2}\right)f_{y}}{\pi^{2}{t_{w}}^{2}E}}$$

The plastic horizontal shear resistance of the web post computed as given in Eq. (23) and related to the vertical shear resistance (Vv) capacity of upper and lower tees as given in Eq. (22).

**Fig. 15:**
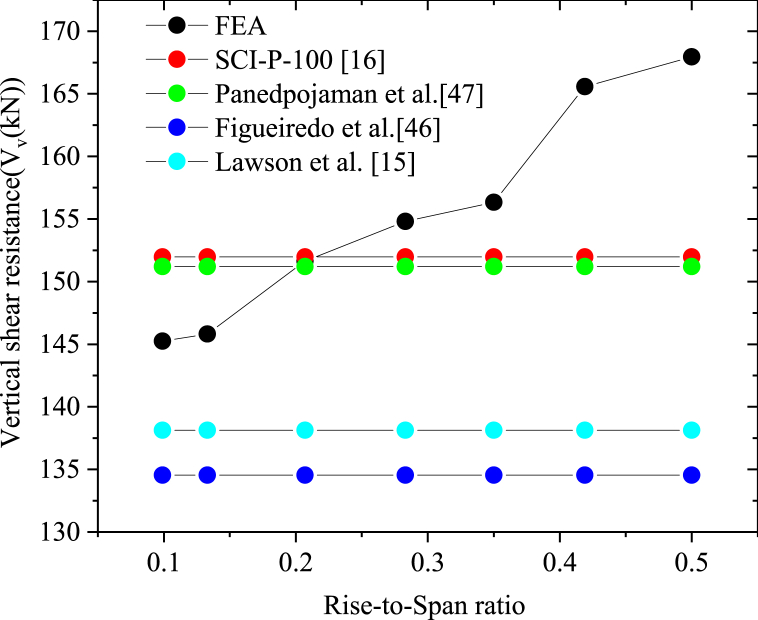
Isolated arched web vertical shear resistance comparison of different analysing approaches.

From the finite element analysis results of isolated arched web post, it was found that the ultimate shear resistance of web post also increases with the rise-to-span ratio as observed in full span arched cellular steel beams as shown in Table 9 and Fig. 15. The finite element analysis of isolated arched web post shear resistance shows that designing web post shear resistance of arched web post according to BS EN 1995-1-1 of a member under compression along with analytical equation proposed for effective buckling length by Lawson et al. [15] underestimate arched web post shear resistance as depicted in Fig. 15. In addition, the analytical approach proposed by Figueiredo et al. [45] to determine the web post shear resistance yields, lower web post-buckling shear resistance than the arched web post shear resistance as shown in Table 9 and Fig. 15. However, these two analytical approaches give conservative and structurally safe results in determining arched web post ultimate shear resistance irrespective of the rise-to-span ratio as depicted in Fig. 15. On the other hand, the web post shear resistance approaches proposed by Panedpojaman et al. [46] and SCI P-100 [16] overestimate the web post shear resistance of shallow arched web post of low rise-to-span ratio. But as the rise-to-span ratio of arch increases, the Panedpojman et al. [46] and the SCI-100 [16] web post shear resistance analyzing approaches provides effective shear resistance capacity of arched web post as compared to the Lawson et al. [15] and Figueiredo et al. [45] Web post shear resistance analyzing approaches for deep arched cellular steel beam, as it can be seen in Fig. 15.

**Table 9:** FEA results of isolated arched web post vertical shear resistance in comparison to different web post shear resistance analysing model.

Models	Rise-to-span ratio	FEA	SCI–P-100 [16]	Lawson et al. [15]	Panedpojaman et al. [46]	Figueiredo et al. [45]
SCC-1	0.099	145.25	151.97	138.13	151.2	134.54
SCC-2	0.133	145.81	151.97	138.13	151.2	134.54
SCC-3	0.207	151.63	151.97	138.13	151.2	134.54
DCC-1	0.283	154.81	151.97	138.13	151.2	134.54
DCC-2	0.35	156.34	151.97	138.13	151.2	134.54
DCC-3	0.419	165.57	151.97	138.13	151.2	134.54
DCC-4	0.5	167.96	151.97	138.13	151.12	134.54

**Fig. 16:**
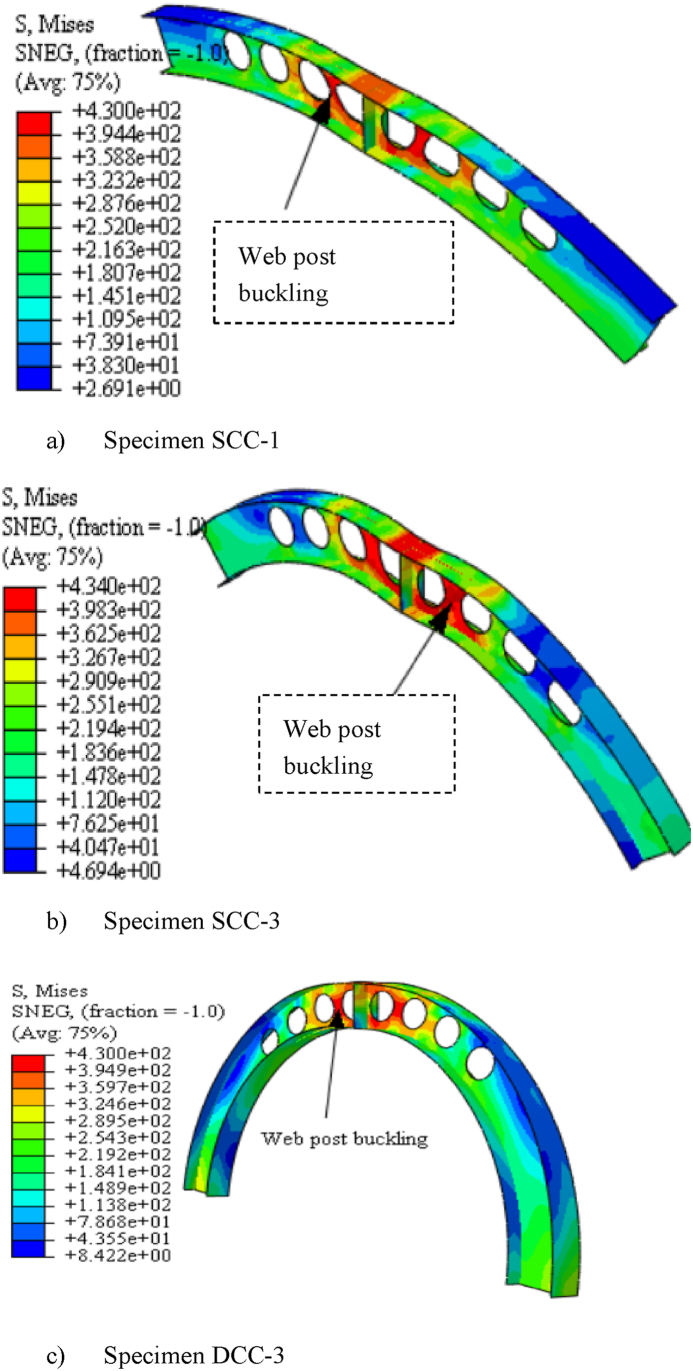
Web post-buckling deformation under mid-span point load.

**Fig. 17:**
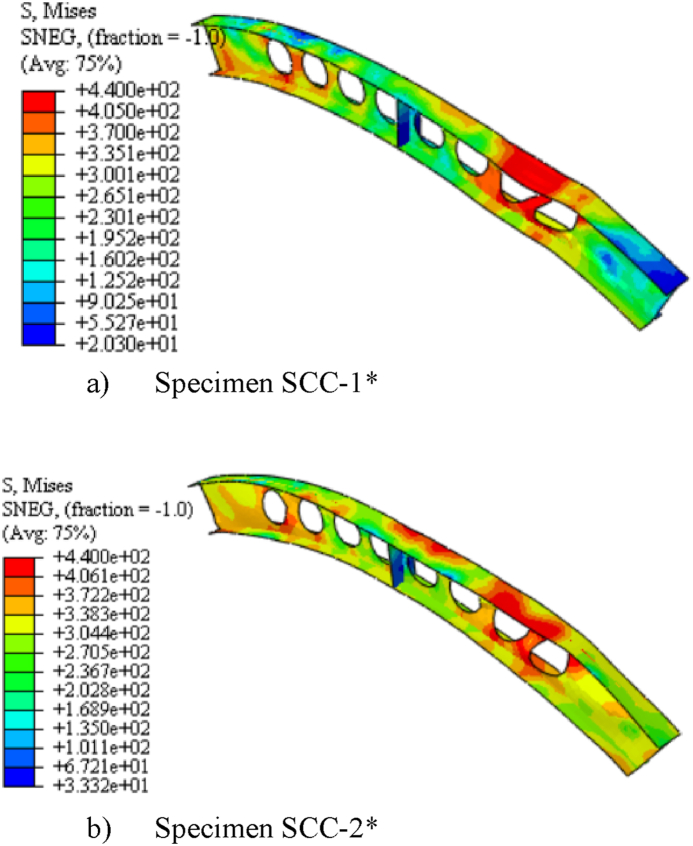
Web post lateral torsional buckling.

#### 3.4.2 Out -of- plane buckling

**Fig. 18:**
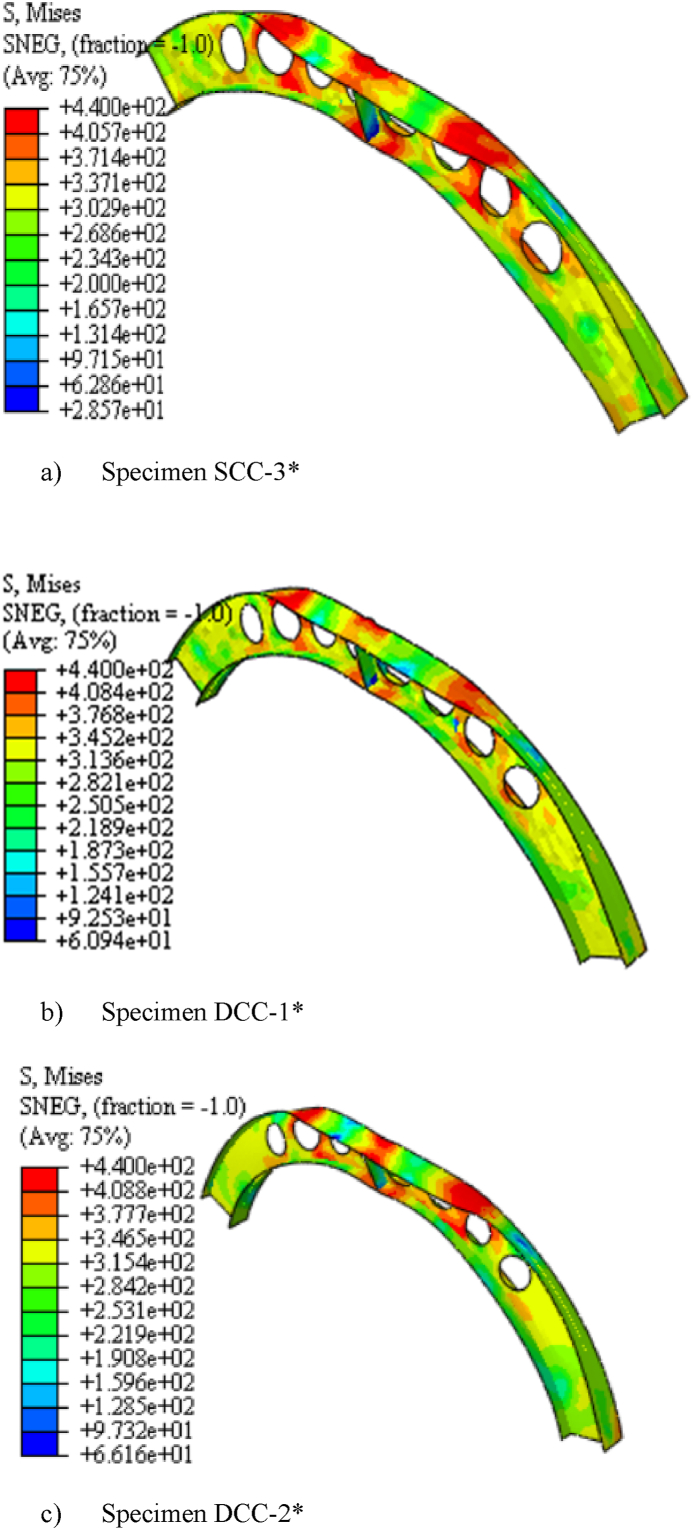
In -elastic symmetric out of plane deformation.

**Fig. 19:**
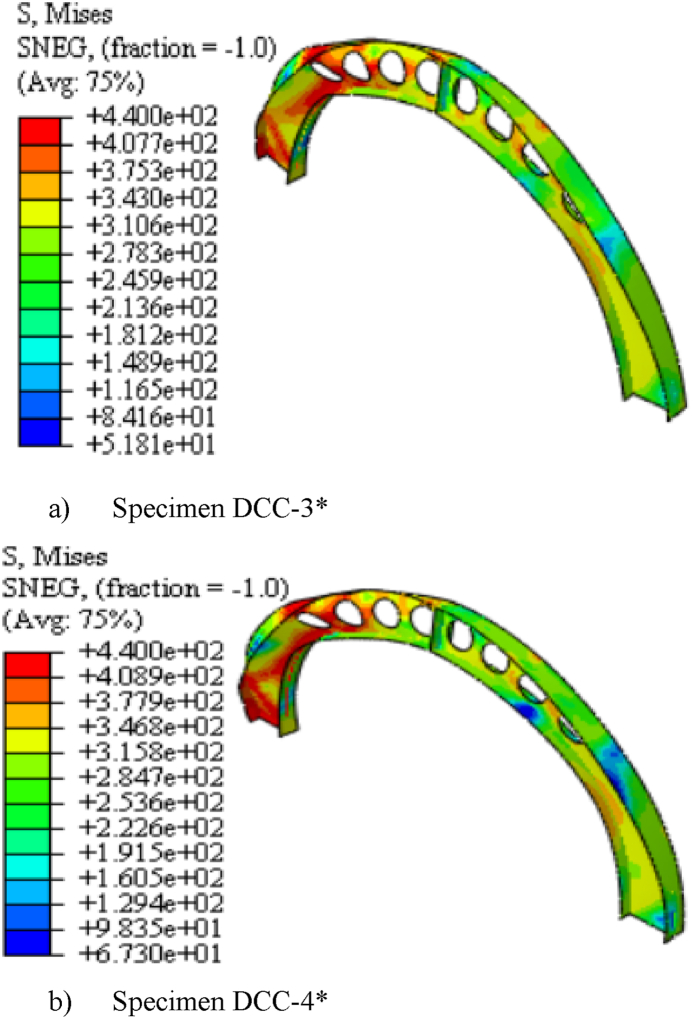
Unsymmetrical out of plane buckling mode.

From the three-dimensional finite element analysis results, under uniform radially distributed vertical load, different out-of-plane buckling modes were observed depending on the arch degree of curvature. Shallow arch SCC-1* and SCC-2* specimens buckle in unsymmetrical web post lateral torsional buckling manner as shown in Fig. 17 (a)–(b). Specimens SCC-3*, DCC-1*, and DCC-2* buckle in similar symmetric out-of-plane buckling mode as revealed in Fig. 18 (a)-(b). The load versus out-of-plane deflection curve of the test samples were depicted in Fig. 7 (d). While, extremely deep arch specimens DCC-3* and DCC-4* failed in unsymmetrical out-of-plane buckling mode as shown in Fig. 19 (a)-(b). The observed failure modes generally indicate that the combined non-uniform axial compressive, twisting, and bending internal action is induced in the span of the arched cellular steel beams under applied uniformly distributed vertical loads. The distribution of this induced stress altered as the arch rise-to-span ratio varied. Because of this, circular arched cellular steel beams with varied rise-to-span ratios, but the same span length may buckle in different ways when subjected to uniformly distributed vertical loads.

**Fig. 20:**
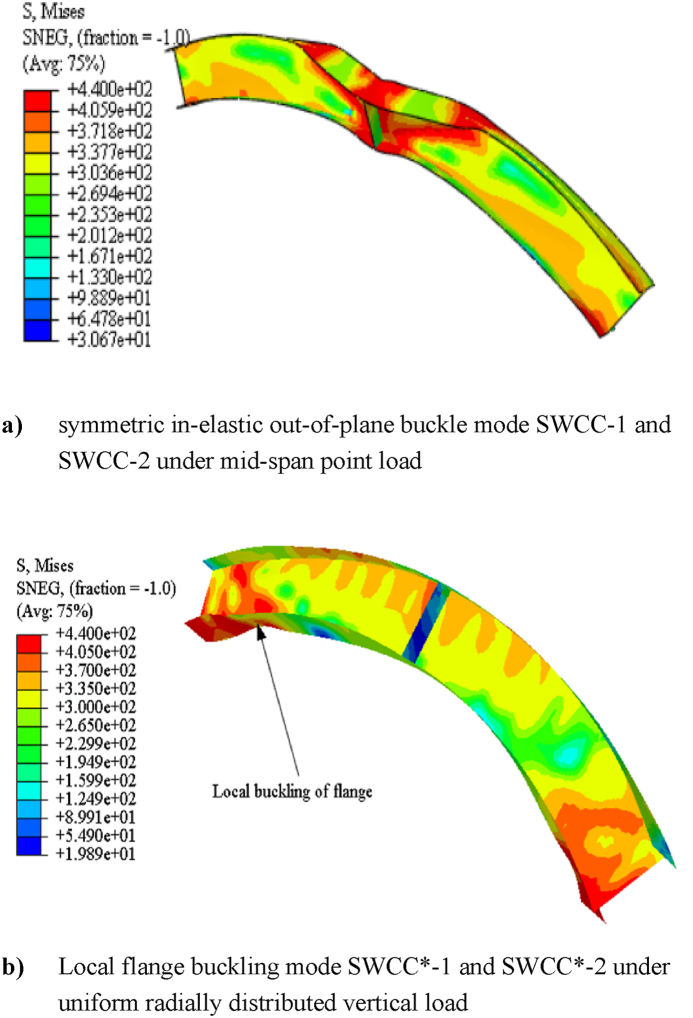
Buckling mode of solid web shallow circular steel arch.

**Fig. 21:**
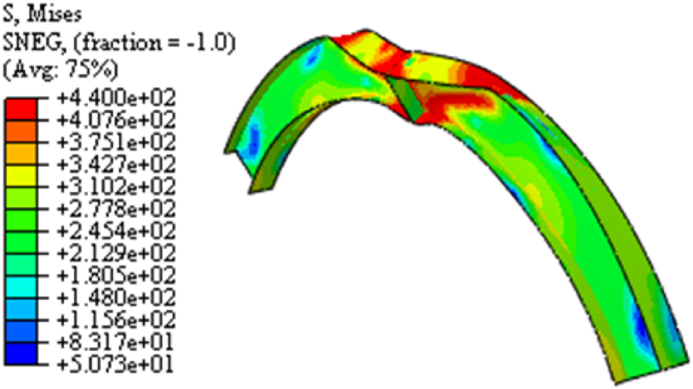
In-elastic symmetric out-of-plane buckling of SWDCC-1 and SWDCC-2 under mid-span point.

**Fig. 22:**
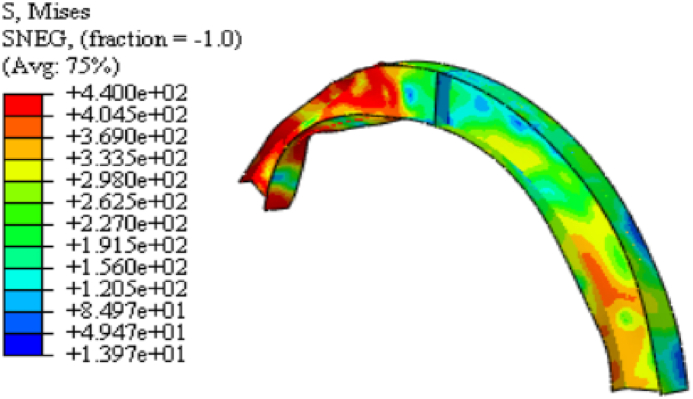
Unsymmetrical out of plane buckling mode of SWDCC-4.

## 4 Conclusions

This paper presents a numerical investigation of the in-plane elastic-plastic complex effect of a rise-to-span ratio, radius of curvature, impacts of the web opening with associated post-buckling failure mode, and arched web post shear resistance of circular arched cellular steel beam using a nonlinear finite element program ABAQUS. The investigation performed on arches ranges from the shallow to deep arches depending on a subtended angle under mid-span point load and uniform radially distributed vertical load. Based on this study, the following conclusions were drawn.

• The proposed finite element analysis method and procedure replicates the experimental work to determine ultimate inelastic buckling load, web post shear resistance of arched web post, and post-buckling behavior of arched cellular steel beams.
• The web post shear determining analytical model proposed by Lawson et al. [15] and Figueiredo et al. [45] Provides conservative results when compared to the FEA results of arched web post shear resistance as the rise-to-span ratio of the arch increases. The web post shear resistance analyzing approaches proposed by Panedpojaman et al. [46] and SCI–P-100 [16] provides a structurally unsafe prediction (overestimate) the shear resistance of arched web post for low rise-to-span ratio arched cellular steel beams.
• Under concentrated mid-span point load, the inelastic buckling load carrying capacity of circular arched cellular steel beam increases with the increasing the rise-to-span ratio.
• The inelastic buckling load carrying capacity of circular arched steel beam increases with the increase of the rise-to-span ratio up to 0.35 or subtended angle of 140° under uniform radially distributed vertical load. An increase in the rise-to-span ratio beyond 0.35 or subtended angel 140° reduces the structural stiffness of the arch.
• Increasing the radius of curvature without altering the rise-to-span ratio and subtended angle reduces the ultimate buckling load-carrying capacity of circular axis arched cellular steel beam.
• The presence of repeated web openings significantly reduces structural stiffness. In addition, the presence of web openings also alters the internal stress distribution and buckling modes.
• Under a uniform distributed vertical load, increasing the radius of curvature also increases the ultimate buckling load of shallow arches. Conversely, increasing the radius of curvature reduces the ultimate buckling load of deep arched circular cellular steel.
• Local web post-buckling failure mode was the common failure mode of arched cellular steel beam under the mid-span concentrated load regardless of arch's rise-to-span ratio.
• Different out-of-plane buckling modes were observed depending upon the arch rise-to-span ratio under uniformly distributed vertical load. Shallow arches in the range of rise-to-span ratio [0.09–0.133] fail in unsymmetrical lateral torsional web post-buckling fashion, symmetric out-of-plane buckling was observed in arches of rise-to-span ratio in the range of [0.207–0.35], and unsymmetrical out of plane buckling detected for the very deep arch of rise-to-span ratio [0.401–0.5].

This study presented a detailed nonlinear FEA model and focused on the parametric study on the in-plane elastic-plastic buckling performance of circular arched cellular beam and arched web post shear resistance under mid-span point load and uniform radially distributed vertical loads. But, to comprehensively understand and generalize the structural behavior of arched cellular steel beams, more experimental, analytical, FEA, and machine learning models considering the residual stress and covering the effects of different opening shapes, opening spacing variation, uneven loading conditions and lateral torsional buckling resistance of arched cellular steel beams requires further studies.

## Data availability statement

All the data and model used in this study, presented and referenced in the article. Supplemental data to support further study on the topic will be provided upon request.

## Funding

There is no external funding received for this study.

## CRediT authorship contribution statement

**Besukal Befikadu Zewudie:** Conceptualization, Data curation, Formal analysis, Investigation, Methodology, Software, Validation, Visualization, Writing – original draft, Writing – review & editing. **Kefiyalew Zerfu:** Data curation, Resources, Supervision, Writing – review & editing. **Elmer C. Agon:** Resources, Supervision, Writing – review & editing.

## Declaration of competing interest

The authors declare that they have no known competing financial interests or personal relationship that could have appeared to influence the work reported in this paper.
